# Combined endoscopic submucosal dissection and endoscopic laryngopharyngeal surgery for an oropharyngeal lesion extending to the flap of a glossopharyngeal reconstruction

**DOI:** 10.1055/a-2462-1144

**Published:** 2024-11-26

**Authors:** Kotaro Waki, Kenshi Matsuno, Hideaki Miyamoto, Ryosuke Gushima, Daizo Murakami, Yorihisa Orita, Yasuhito Tanaka

**Affiliations:** 1 13205 Gastroenterology and Hepatology, Faculty of Life Sciences, Kumamoto University, Kumamoto, Japan; 2 157728 Otolaryngology-Head and Neck Surgery, Kumamoto University Hospital, Kumamoto, Japan

Pharyngeal endoscopic submucosal dissection (ESD) 1 and endoscopic laryngopharyngeal surgery (ELPS) 2 3 are reportedly effective treatments for superficial pharyngeal cancer (SPC). ESD allows the clinician to perform highly precise procedures involving large treatable areas owing to endoscopic manipulation. Although the treatable area of ELPS is limited by the mobility of the transoral devices, the operator can independently control the traction during dissection, resulting in efficient procedures 3 . Considering these characteristics, we present a case of SPC extending to the reconstructed flap that was resected using a combined ESD and ELPS procedure.

A 56-year-old man who had undergone total glossectomy and laryngectomy with glossopharyngeal reconstruction using the pectoralis major myocutaneous flap for oropharyngeal cancer 10 years previously underwent surveillance esophagogastroduodenoscopy. A 40-mm SPC was found on the posterior wall of the oropharynx extending to the reconstructed flap ( Fig. 1 ). ESD and ELPS were therefore planned and performed ( Video 1 ).

**Fig. 1:**
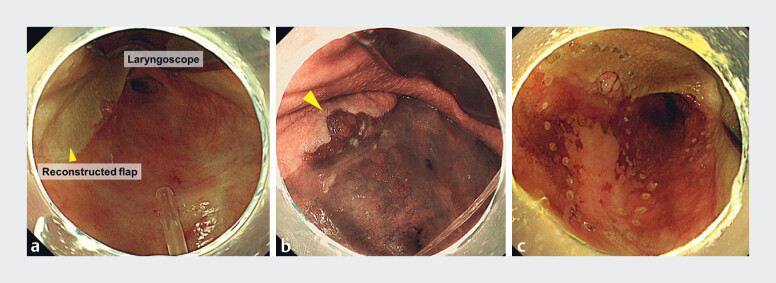
Endoscopic images of the 40-mm lesion on the posterior wall of the oropharynx showing: **a** on distant view, the left wall to anterior wall of the oropharynx that was reconstructed using a left pectoralis major myocutaneous flap and the laryngoscope in position; **b** on close-up view, the part of the lesion that was extending to the flap (yellow arrowhead); **c** the appearance after marking had been performed using magnification with narrow-band imaging and iodine staining.

Combined endoscopic submucosal dissection and endoscopic laryngopharyngeal surgery are performed for an oropharyngeal lesion extending to the reconstructed flap.Video 1

We first made an incision and dissected from the oral side to the flap side using ESD with a DualKnife J (KD-655Q; Olympus, Tokyo, Japan) ( Fig. 2 ), so that the edge of the specimen could be easily grasped with forceps. Performing ELPS with a Colorado MicroDissection Needle (E103; Stryker, Portage, Michigan, USA), we then performed a dissection focusing on the subcutaneous tissue layer, which contained a significant amount of fat. Afterward, we switched between ESD with/without traction 4 and ELPS as needed, achieving en bloc resection within 67 minutes, with no adverse events ( Fig. 3 ). Histologic examination revealed a subepithelial invasive squamous cell carcinoma with negative margins.

**Fig. 2:**
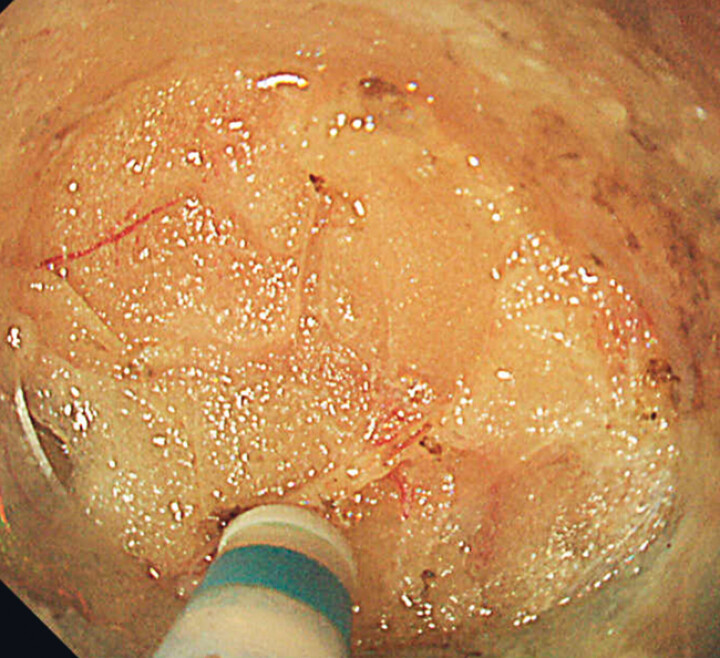
Endoscopic image during dissection of the flap, with a significant amount of fat visible within the subcutaneous tissue.

**Fig. 3:**
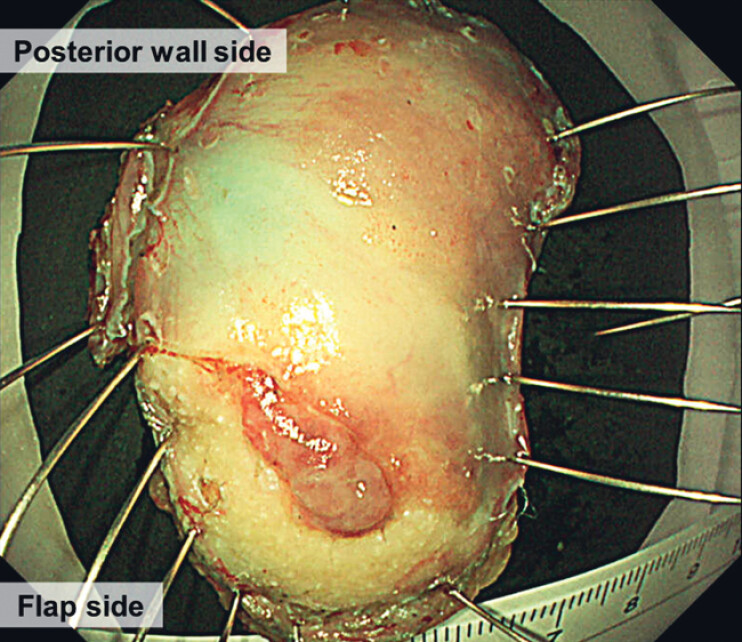
Macroscopic appearance of the resected specimen, which was shown histologically to be a squamous cell carcinoma (0.7-mm thick) with negative margins.

Although there has been a previous case report of a lesion on the flap that was treated with ESD alone 5 , resecting the layer of fat using only this technique is not efficient. We believe that the combination of ESD and ELPS is one option that could be used for the efficient treatment of lesions on the flap.

Endoscopy_UCTN_Code_TTT_1AO_2AG_3AD

## Notes

Endoscopy E-Videos https://eref.thieme.de/e-videos
*E-Videos* is an open access online section of the journal *Endoscopy* , reporting on interesting cases and new techniques in gastroenterological endoscopy. All papers include a high-quality video and are published with a Creative Commons CC-BY license. Endoscopy E-Videos qualify for HINARI discounts and waivers and eligibility is automatically checked during the submission process. We grant 100% waivers to articles whose corresponding authors are based in Group A countries and 50% waivers to those who are based in Group B countries as classified by Research4Life (see: https://www.research4life.org/access/eligibility/ ). This section has its own submission website at https://mc.manuscriptcentral.com/e-videos .
